# Co-production of hydrogen and ethanol from glucose in *Escherichia coli* by activation of pentose-phosphate pathway through deletion of phosphoglucose isomerase (*pgi*) and overexpression of glucose-6-phosphate dehydrogenase (*zwf*) and 6-phosphogluconate dehydrogenase (*gnd*)

**DOI:** 10.1186/s13068-017-0768-2

**Published:** 2017-03-29

**Authors:** Balaji Sundara Sekar, Eunhee Seol, Sunghoon Park

**Affiliations:** Department of Chemical and Biomolecular Engineering, Pusan National University, 2, Busandaehak-ro 63 beon-gil, Geumjeong-gu, Busan, 46241 Republic of Korea

**Keywords:** Biohydrogen, Co-production of hydrogen and ethanol, Phosphoglucose isomerase deletion, Pentose-phosphate pathway, *Escherichia coli*

## Abstract

**Background:**

Biologically, hydrogen (H_2_) can be produced through dark fermentation and photofermentation. Dark fermentation is fast in rate and simple in reactor design, but H_2_ production yield is unsatisfactorily low as <4 mol H_2_/mol glucose. To address this challenge, simultaneous production of H_2_ and ethanol has been suggested. Co-production of ethanol and H_2_ requires enhanced formation of NAD(P)H during catabolism of glucose, which can be accomplished by diversion of glycolytic flux from the Embden–Meyerhof–Parnas (EMP) pathway to the pentose-phosphate (PP) pathway in *Escherichia coli*. However, the disruption of *pgi* (*p*hospho*g*lucose *i*somerase) for complete diversion of carbon flux to the PP pathway made *E. coli* unable to grow on glucose under anaerobic condition.

**Results:**

Here, we demonstrate that, when glucose-6-phosphate dehydrogenase (Zwf) and 6-phosphogluconate dehydrogenase (Gnd), two major enzymes of the PP pathway, are homologously overexpressed, *E. coli* Δ*pgi* can recover its anaerobic growth capability on glucose. Further, with additional deletions of Δ*hycA*, Δ*hyaAB*, Δ*hybBC*, Δ*ldhA*, and Δ*frdAB*, the recombinant Δ*pgi* mutant could produce 1.69 mol H_2_ and 1.50 mol ethanol from 1 mol glucose. However, acetate was produced at 0.18 mol mol^−1^ glucose, indicating that some carbon is metabolized through the Entner–Doudoroff (ED) pathway. To further improve the flux via the PP pathway, heterologous *zwf* and *gnd* from *Leuconostoc mesenteroides* and *Gluconobacter oxydans*, respectively, which are less inhibited by NADPH, were overexpressed. The new recombinant produced more ethanol at 1.62 mol mol^−1^ glucose along with 1.74 mol H_2_ mol^−1^ glucose, which are close to the theoretically maximal yields, 1.67 mol mol^−1^ each for ethanol and H_2_. However, the attempt to delete the ED pathway in the Δ*pgi* mutant to operate the PP pathway as the sole glycolytic route, was unsuccessful.

**Conclusions:**

By deletion of *pgi* and overexpression of heterologous *zwf* and *gnd* in *E. coli* Δ*hycA* Δ*hyaAB* Δ*hybBC* Δ*ldhA* Δ*frdAB*, two important biofuels, ethanol and H_2_, could be successfully co-produced at high yields close to their theoretical maximums. The strains developed in this study should be applicable for the production of other biofuels and biochemicals, which requires supply of excessive reducing power under anaerobic conditions.

**Electronic supplementary material:**

The online version of this article (doi:10.1186/s13068-017-0768-2) contains supplementary material, which is available to authorized users.

## Background

Hydrogen (H_2_) is considered as a promising alternative to fossil fuel as it is an efficient energy carrier and produces zero carbon emission. Currently, H_2_ is produced by steam reforming process using natural gas, a non-renewable source. Therefore, as an alternative, biological H_2_ production by photolysis, photofermentation, or dark fermentation has been studied for decades due to its dependence on renewable energy source. Dark fermentation, owing to its rapidity and simplicity, usually is the preferred approach [1–3], though its theoretical H_2_ yield is low: typically <2 mol mol^−1^ glucose for mesophilic bacteria such as *Escherichia coli* [4] and <4 mol mol^−1^ glucose for strict anaerobes such as *Clostridia*, *Thermoanaerobacter tengcongensis*, *Pyrococcus furiosus*, and others [5–7]. To improve H_2_ production yield in *E. coli*, introduction of heterologous pathways such as ferredoxin- or NAD(P)H-dependent H_2_ production pathways has been attempted. The heterologous pathways, though functional in *E. coli*, have been shown to be highly inefficient and, as such, non-conducive to practical improvements in H_2_ yield [8]. In the case of strict anaerobes, higher yield, close to 4 mol mol^−1^ glucose, have been reported [9], albeit still not high enough to be commercially interesting. Due to the lack of a genetic tool box and/or the difficulty for gene manipulation, serious pathway engineering in strict anaerobes is yet to be attempted. As alternative means of addressing dark fermentation’s low H_2_ production yield, hybrid processes such as dark *plus* photofermentation, hythane process (production of H_2_ in the first stage and methane in the second), etc., have been studied [10–12]. Albeit efficient and feasible on the laboratory scale, these hybrid processes’ industrial application is highly challenging due to complicated reactor configurations and/or operation. We have suggested, as an alternative, a simple process by which H_2_ and ethanol are co-produced in a single bioreactor [13]. Ethanol in fact is a good liquid biofuel, and can easily be separated from gaseous H_2_. Co-production of ethanol with H_2_, moreover, can significantly increase the energy recovery of dark fermentation and, thereby, make H_2_ production from glucose more attractive [14].

**Fig. 1:**
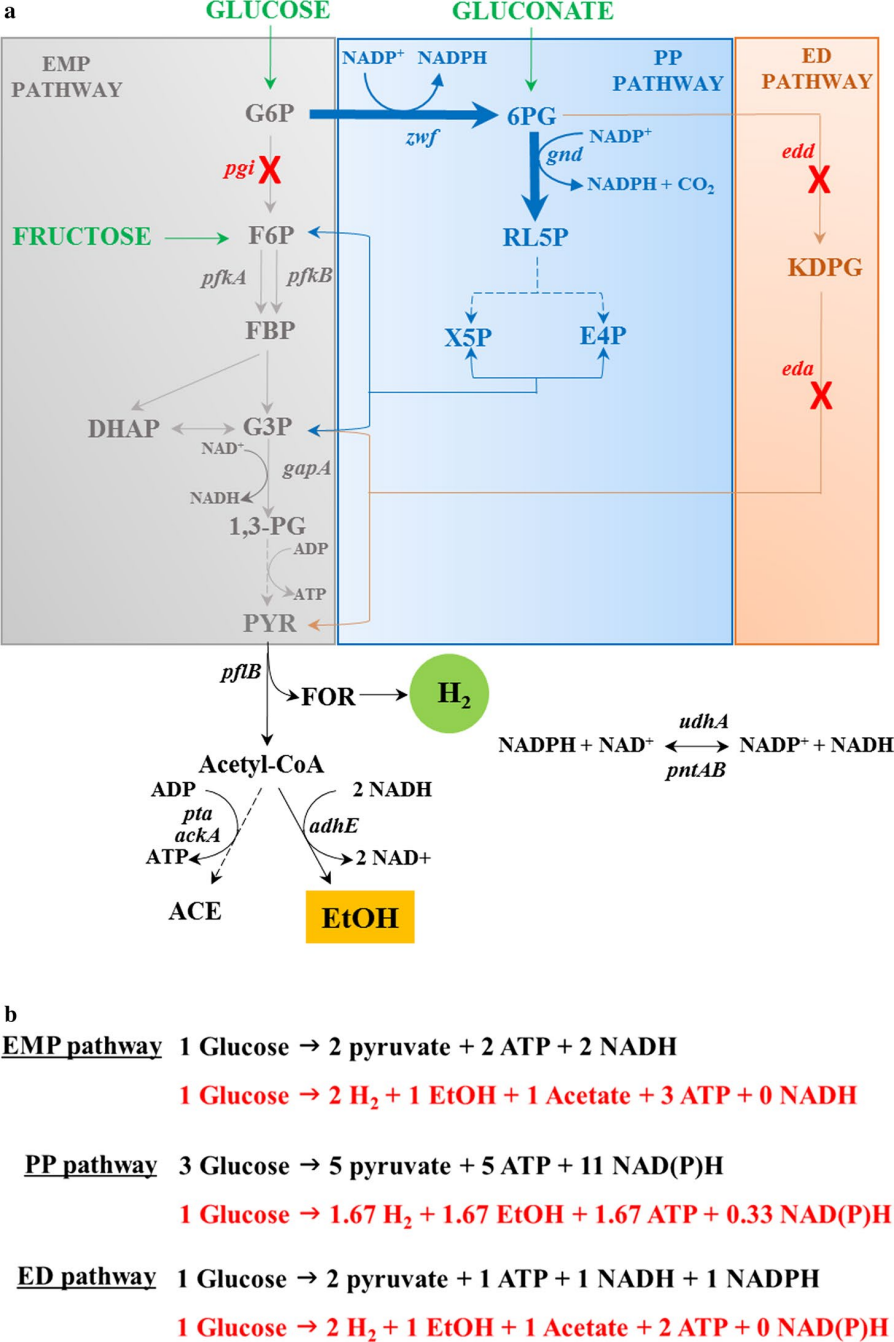
**a** Pathway engineering for promotion of carbon flux through PP pathway. The EMP and ED pathways were disrupted by deleting *pgi*, *edd,* and *eda* (*red crosses*), and the PP pathway was activated by the overexpression of *zwf* and *gnd* (*bold blue arrows*). **b** Theoretical carbon and energy balance of EMP, PP (non-cyclic), and ED pathway of base strain (SH5) for co-production of H_2_ and ethanol. Genes: *pgi*—phosphoglucose isomerase, *pfk*—phosphofructokinase, *gapA*—glyceraldehyde-3-phosphate dehydrogenase, *pta*—phosphotransacetylase, *ackA*—acetate kinase, *adhE*—alcohol dehydrogenase, *zwf*—glucose-6-phosphate dehydrogenase, *gnd*—6-phosphogluconate dehydrogenase, *edd*—Entner–Doudoroff dehydratase, *eda*—Entner–Doudoroff aldolase, *udhA*—soluble transhydrogenase, *pntAB*—membrane-bound transhydrogenase. Metabolites: G6P—Glucose-6-phosphate, F6P—fructose-6-phosphate, FBP—fructose-1,6-bisphosphate, DHAP—dihydroxyacetone phosphate, G3P—glyceraldehyde-3-phosphate, 1,3-PG—1,3-bisphosphoglycerate, PYR—pyruvate, FOR—formate, H_2_—hydrogen, AcCoA—acetyl-CoA, ACE—acetate, EtOH—ethanol, 6PG—6-phosphogluconate, RL5P—ribulose-5-phosphate, X5P—xylose-5-phosphate, E4P—erythrose-4-phosphate, KDPG—2-Keto-3-deoxy-6-phosphogluconate

Under typical anaerobic conditions, *E. coli* metabolizes most of the glucose through the EMP pathway, producing H_2_ at 2 mol mol^−1^ glucose along with ethanol and acetate each at 1 mol mol^−1^ glucose (Fig. 1). Co-production of ethanol with H_2_ requires the production of ethanol instead of acetate, which entails conversion of the two molecules of acetyl-CoA produced from one molecule of glucose to two molecules of ethanol, without producing acetate. This requires additional NAD(P)H generation during glycolysis. For this purpose, use of a more reduced carbon source than glucose, such as glycerol, has been studied [15] for the production of equimolar H_2_ and ethanol without acetate. However, with glucose as the carbon source, the theoretical maximum yield of ethanol cannot exceed 1 mol per mol of glucose, because the EMP pathway generates only 2 mol NADH per mol of glucose. However, if glucose is exclusively metabolized through the PP pathway, 3.67 mol NAD(P)H per mol of glucose can be generated, which, in theory, enables co-production of ethanol and H_2_, each at 1.67 mol mol^−1^, without acetate [13]. To this end, blockage of the EMP pathway by deletion of phosphoglucose isomerase (Pgi) has been attempted; unfortunately though, the Δ*pgi* strain could not grow anaerobically. In response, for co-production of H_2_ and ethanol, alternative, phosphofructokinase-1 (PfkA) deletion mutants have been developed and studied (Fig. 1) [14, 16]. The Δ*pfkA* strains could grow well after long adaptation to anaerobic conditions [14] and produced significant amounts of H_2_ and ethanol (~1.8 mol H_2_ mol^−1^ and ~1.40 mol ethanol mol^−1^) when Zwf and Gnd, the key enzymes of the PP pathway, were overexpressed. However, due to the active expression of *pfkB*, which encodes for isozyme of PfkA, up to 30% of the glucose was metabolized through the EMP pathway in the Δ*pfkA* strains, thus resulting in substantial acetate production (~0.15 mol mol^−1^) [14].

In the present study, we determined that Δ*pgi* mutants can grow anaerobically when Zwf and Gnd are overexpressed. Consequently, we investigated complete blockage of the carbon flux through the EMP pathway and the concomitantly improved co-production of H_2_ and ethanol. Also, to address the two major disadvantages of Zwf and Gnd of *E. coli*—their dependence on NADP^+^ as the cofactor and serious inhibition of their activities by NADPH at the enzyme level—expressions of heterologous Zwf and Gnd from other microorganisms have been studied. We also attempted deletion of the ED pathway for operation of the PP pathway as the sole glycolytic route. Our results demonstrate that Δ*pgi* mutants can grow under anaerobic conditions and co-produce H_2_ and ethanol at near-theoretical yields. Additionally, the data obtained show that the developed strains can be used as an interesting platform when generation of considerable reducing power is needed in anaerobic glucose metabolism [17, 18].

## Methods

### Strains, plasmids, and materials

The *Escherichia coli* BW25113 mutant strain (SH5) from our previous study [19] was used as a base strain in this work. Restriction and DNA-modifying enzymes were obtained from New England Bio-Labs (Beverly, MA, USA). The Miniprep and DNA gel extraction kits were purchased from Qiagen (Mannheim, Germany). The primers were synthesized by Macrogen Inc. (Seoul, Korea). The yeast extract (Cat. 212750) and Bacto™ tryptone (Cat. 211705) were acquired from Difco (Becton–Dickinson; Franklin Lakes, NJ, USA). Unless indicated otherwise, all of the other chemicals were obtained from Sigma-Aldrich (St. Louis, MO, USA).

### Construction of recombinant *E. coli* strains

For overexpression of Zwf and/or Gnd, the plasmids pEcZ, pEcG, and pEcZG from our previous study were utilized [14]. Gene deletion was performed using either the λ-Red recombinase (deletion of *pgi*) or pKOV system (deletion of *edd*, *udhA*, and *pntAB*) [20, 21]. Briefly, for deletion using λ-Red recombinase, hybrid complementary primers for *pgi* of *E. coli* BW25113 and the antibiotic cassette (FRT-kan-FRT) in pKD4, were used. The amplified FRT-kan-FRT cassette was inserted to SH5 harboring pKD46 plasmid by electroporation. The resulting kanamycin-resistant *E. coli* SH5 containing FRT-kan-FRT in the *pgi* region was isolated using antibiotic resistance screening and PCR with the locus-specific primers. For deletion using pKOV system, recombinant pKOV plasmid was made with PCR-amplified upstream and downstream 500 bp of the target gene. This recombinant plasmid was used to perform double recombination and remove the target gene using sucrose (*sacB*-dependent) as selection pressure. The heterologous *zwf* and *gnd* were codon optimized and synthesized by GenScript (NJ, USA) (Additional file 1: Table S1). All the genes for overexpression were cloned in pDK7 vector and overexpressed under the control of the *tac* promoter [22]. The list of strains constructed in this study is provided in Table 1.

**Table 1:** Strains and plasmids used in this study

	Description	References
Strains		
SH5	BW25113 *ΔhycA ΔhyaAB ΔhybBC ΔldhA ΔfrdAB*	Kim et al. [19]
SH5Δ*pgi*	SH5Δ*pgi*	This study
SH5Δ*pgi*_*Z*	SH5Δ*pgi* harboring pEcZ	
SH5Δ*pgi*_*ZG*	SH5Δ*pgi* harboring p*Ec*ZG	
SH5Δ*pgi*_*ZGU*	SH5Δ*pgi* harboring p*Ec*ZGU	
SH5Δ*pgi*_*Z*_*L*_ *G*_*E*_	SH5Δ*pgi* harboring pLmZ-EcG	
SH5Δ*pgi*_*Z*_*Z*_ *G*_*E*_	SH5Δ*pgi* harboring pZmZ-EcG	
SH5Δ*pgi*_*Z*_*E*_ *G*_*C*_	SH5Δ*pgi* harboring pEcZ-CgG	
SH5Δ*pgi*_*Z*_*L*_ *G*_*C*_	SH5Δ*pgi* harboring pLmZ-CgG	
SH5Δ*pgi*_*Z*_*Z*_ *G*_*C*_	SH5Δ*pgi* harboring pZmZ-CgG	
SH5Δ*pgi*_*Z*_*E*_ *G*_*G*_	SH5Δ*pgi* harboring pEcZ-GoG	
SH5Δ*pgi*_*Z*_*L*_ *G*_*G*_	SH5Δ*pgi* harboring pLmZ-GoG	
SH5Δ*pgi*_*Z*_*Z*_ *G*_*G*_	SH5Δ*pgi* harboring pZmZ-GoG	
SH5Δ*pgi*Δ*edd*	SH5Δ*pgi* Δ*eddeda*	
SH5Δ*pgi*Δ*edd*_*G*	SH5Δ*pgi*Δ*edd* harboring p*Ec*G	
SH5Δ*pgi*Δ*edd*_*ZG*	SH5Δ*pgi*Δ*edd* harboring p*Ec*ZG	
SH5Δ*pgi*Δ*edd*_*Z*_*L*_ *G*_*G*_	SH5Δ*pgi*Δ*edd* harboring pLmZ-GoG	
Plasmids		
pDK7	Expression vector	Kleiner et al. [22]
pEcZ	pDK7 carrying *zwf* of *E. coli* BW25113	Sundara Sekar et al. [14]
pEcZG	pDK7 carrying *zwf, gnd* of *E. coli* BW25113	
pEcZGU	pDK7 carrying *zwf, gnd, udh*A of *E. coli* BW25113	This study
pLmZ-EcG	pDK7 carrying *zwf* of *L. mesenteroides* and *gnd* of *E. coli* BW25113	
pZmZ-EcG	pDK7 carrying *zwf* of *Z. mobilis* and *gnd* of *E. coli* BW25113	
pEcZ-CgG	pDK7 carrying *zwf* of *E. coli* BW25113 and *gnd* of *C. glutamicum*	
pLmZ-CgG	pDK7 carrying *zwf* of *L. mesenteroides* and *gnd* of *C. glutamicum*	
pZmZ-CgG	pDK7 carrying *zwf* of *Z. mobilis* and *gnd* of *C. glutamicum*	
pEcZ-GoG	pDK7 carrying *zwf* of *E. coli* BW25113 and *gnd* of *G. oxydans*	
pLmZ-GoG	pDK7 carrying *zwf* of *L. mesenteroides* and *gnd* of *G. oxydans*	
pZmZ-GoG	pDK7 carrying *zwf* of *Z. mobilis* and *gnd* of *G. oxydans*	

### Culture conditions

Luria-Bertani broth was used to culture the cells for genetic engineering and culture maintenance work. Production studies were performed in modified M9 medium containing 5.0 g L^−1^ glucose or gluconate, 1.0 g L^−1^ yeast extract, 3.0 g L^−1^ Na_2_HPO_4_, 1.5 g L^−1^ KH_2_PO_4_, 0.5 g L^−1^ NH_4_Cl, 0.25 g L^−1^ NaCl, 0.25 g L^−1^ MgSO_4_, and 0.01 g L^−1^ CaCl_2_. Kanamycin (50 µg mL^−1^) and chloramphenicol (25 µg mL^−1^) were added to the medium for culturing of the recombinant strains. The medium was also supplemented with 0.2 mg L^−1^ NiSO_4_, 1.4 mg L^−1^ FeSO_4_, 0.2 mg L^−1^ Na_2_SeO_3_, 0.2 mg L^−1^ Na_2_MoO_4_, and 8.8 mg L^−1^ cysteine HCl for supporting the synthesis of co-production-related enzymes. The cells were cultured anaerobically with 50 mL of M9 medium in 165 mL serum bottles. The serum bottles with the media were flushed with argon for 15 min to create the anoxic condition for fermentation. The cells were cultured at 37 °C in an orbital shaker rotating at 200 rpm. The expressions of Zwf and Gnd were induced by the addition of 0.1 mM isopropyl-β-d-thiogalactopyranoside (IPTG), unless stated otherwise.

### Total RNA isolation and real-time PCR

The recombinant strains were induced with IPTG and harvested during the late exponential growth phase. RNAprotect reagent (Qiagen Inc., USA) was added to the cell pellets, which were stored at −80 °C to prevent RNA degradation. Total RNA was extracted using the Nucleospin^®^ RNA isolation kit (Macherey-Nagel, Germany) and converted to cDNA using the SuperScript III first-strand synthesis system (Invitrogen, USA). The RT-PCR primers were designed using Primer Express^®^ software. RT-PCR analysis was performed using the StepOne real-time PCR system (Applied Biosystems, USA). The experiment was conducted in duplicate using the SYBR Green method, and the relative mRNA was quantified by the ΔC_T_ method [23]. *rpoD* was utilized as the endogenous control.

### Determination of enzyme activities

The enzyme activities of Gnd and Zwf were measured as described in Moritz et al. [24], with slight modifications. Briefly, the enzyme activities were performed in 50 mM Tris-HCl (pH 7.5) containing 0.2 mM NADP^+^, 1 mM MgCl_2_, and 0.5 mM glucose-6-phosphate or 6-phosphogluconate. The reduction of NADP^+^ was observed at 340 nm. The extinction coefficient (*ε*_340_) of 6.22 mM^−1^ cm^−1^ was used for calculating the amount of NADPH formed in the assay. All measurements were performed at 30 °C.

### Analytical methods

Cell growth was monitored by UV spectrophotometry (Lambda 20, Perkin Elmer, USA) measurement of the optical density (OD_600_) at 600 nm. Gases such as H_2_ and CO_2_ were measured by gas chromatography (DS6200 Donam Systems Inc., Seoul, Korea) equipped with a TCD detector. The stainless steel column of gas chromatography was packed with either Hayesep Q (for CO_2_ analysis, Alltech Deerfield, IL, USA) or Molecular Sieve 5A (for H_2_ analysis, Alltech Deerfield, IL, USA). Argon was used as the carrier gas and its flow rate was set at 30 mL min^−1^. The temperature of injector, column oven, and TCD detector was maintained at 90, 80, and 120 °C, respectively, during analysis. Glucose, ethanol, and all of the other metabolites were measured by high-performance liquid chromatography (Agilent Technologies, HP, 1200 series) equipped with a refractive index (RID) and photodiode array (DAD) detectors. The post-fermentation medium was centrifuged and filtered, and samples were eluted through a 300 mm × 7.8 mm Aminex HPX-87H (Bio-Rad) column at 65 °C using 2.5 mM H_2_SO_4_. The protein concentrations of the samples used in the enzyme activities were determined by the Bradford method as described previously [25] using bovine serum albumin as the standard.

## Results and discussion

### Growth of *E. coli* mutant lacking *pgi* under aerobic and anaerobic conditions

**Fig. 2:**
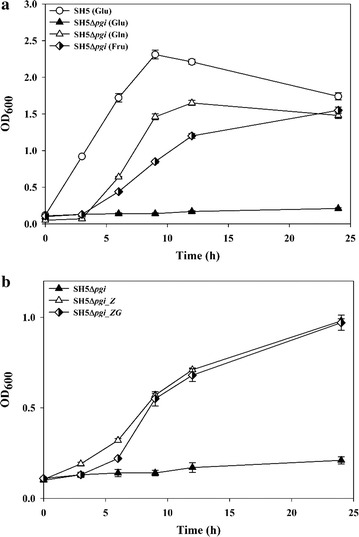
Anaerobic growth of SH5, SH5Δ*pgi*, and recombinant SH5Δ*pgi* strains. **a** Growth of SH5 and SH5Δ*pgi* on different carbon sources such as glucose (Glu), gluconate (Gln), and fructose (Fru). **b** Growth of SH5Δ*pgi*, SH5Δ*pgi*_*Z*, and SH5Δ*pgi*_*ZG* with glucose as carbon source. Refer to Table 1 for the genotype of each strain

To completely block the carbon flux through the EMP pathway and divert it through the PP pathway, the *pgi* gene was deleted from the *E. coli* BW25113 mutant strain (designated as ‘SH5’), which has several deletions in such enzymes as uptake hydrogenases (*hyaAB*, *hybBC*), the negative regulator of formate-hydrogen lyase (*hycA*), lactate dehydrogenase (*ldhA*), and fumarate reductase (*frdAB*) [19]. The resulting mutant strain *E. coli* SH5Δ*pgi* could grow well on glucose under aerobic conditions but not under anaerobic conditions (Fig. 2a). Laboratory adaptive evolution by repeated transfers in glucose medium under anaerobic condition was not successful (Additional file 1: Fig. S1). With fructose or gluconate as the carbon source, however, SH5Δ*pgi* could grow well under both aerobic and anaerobic conditions. Fructose enters the EMP pathway through the fructose-6-phosphate (F6P) node, and gluconate enters the PP/ED pathways through the 6-phosphogluconate (6PG) node (Fig. 1). The growth capability of SH5Δ*pgi* on either fructose or gluconate indicated that both the EMP pathway (after F6P) and the PP/ED pathway (after 6PG) functioned properly. In this light, we hypothesized that the lack of anaerobic growth of SH5Δ*pgi* on glucose was caused by its inability to convert glucose-6-phosphate (G6P) to 6PG. We further posited that, when expressed from the chromosome, the activities of glucose-6-phosphate dehydrogenase (Zwf) and/or 6-phosphogluconolactonase (Pgl), which are the enzymes directing G6P towards the PP or ED pathway, are very low [26].

To confirm our hypothesis, *zwf* was homologously overexpressed by a multi-copy plasmid under the IPTG-inducible *tac* promoter. The resultant recombinant, SH5Δ*pgi_Z* could grow well on glucose under anaerobic conditions, though rather slowly compared with its parental strain SH5 (Fig. 2b). In order to determine if *gnd* expression can further improve cell growth, SH5Δ*pgi_ZG* was developed and tested. No difference from SH5Δ*pgi_Z* was observed. It was concluded that the incapacity of *E. coli* Δ*pgi* for anaerobic growth is rooted in the inefficient conversion of G6P to 6PG.

### Co-production of H_2_ and ethanol by *E. coli Δpgi*

Co-production of H_2_ and ethanol was studied and compared among the strains of SH5, SH5Δ*pgi_Z*, and SH5Δ*pgi*_*ZG* (Table 2). The strains were induced with 0.1 mM IPTG, and the metabolites were analyzed at ~24 h of cultivation, when co-production was highest. SH5Δ*pgi_Z*, as compared with SH5, showed improved production of all three major metabolites (H_2_, ethanol, and acetate); this was attributed to the lower cell growth of SH5Δ*pgi_Z* (see Fig. 2) and the conversion of more glucose carbon to those metabolites. However, contrary to our expectation, the ratio of the production yield of ethanol to acetate did not increase in SH5Δ*pgi_Z* relative to SH5 (Table 2). In fact, if the PP pathway, which generates more NAD(P)H, functions as the major glycolytic pathway in SH5Δ*pgi_Z*, more ethanol and less acetate should be produced. It was expected that the ED pathway, rather than the PP pathway, was activated by the overexpression of *zwf*, which generates the same amount of NAD(P)H as the EMP pathway [13]. In comparison, when both *zwf* and *gnd* were overexpressed (SH5Δ*pgi_ZG*), ethanol production was greatly improved to 1.44 mol mol^−1^ with a concomitant acetate reduction to 0.22 mol mol^−1^. We concluded that whereas the cell growth of the Δ*pgi* mutant can be recovered by the overexpression of *zwf* alone, the activation of the PP pathway requires overexpression of both *zwf* and *gnd*. Subsequent experiments with gluconate as a carbon source re-confirmed the importance of *gnd* overexpression for activation of the PP pathway (Table 2). Ethanol production from gluconate by SH5Δ*pgi_Z* was 0.68 mol mol^−1^, similar to that by SH5, while that by SH5Δ*pgi_ZG* was 0.92 mol mol^−1^.

**Table 2:** Co-production by recombinant SH5Δ*pgi* strains overexpressing Zwf and Gnd

Substrate	Strains	Yields of metabolites (mol mol^−1^)		
		H_2_	Ethanol	Acetate
Glucose	SH5	1.44 ± 0.07	0.79 ± 0.02	0.67 ± 0.04
	SH5Δ*pgi*_*Z*	1.81 ± 0.08	0.90 ± 0.02	0.86 ± 0.03
	SH5Δ*pgi*_*ZG*	1.68 ± 0.06	1.44 ± 0.03	0.22 ± 0.02
Gluconate	SH5	1.72 ± 0.09	0.58 ± 0.01	1.31 ± 0.03
	SH5Δ*pgi*_*Z*	1.70 ± 0.05	0.68 ± 0.01	1.25 ± 0.04
	SH5Δ*pgi*_*ZG*	1.69 ± 0.07	0.92 ± 0.02	0.85 ± 0.02

Yields of metabolites were calculated from three individual experiments

**Fig. 3:**
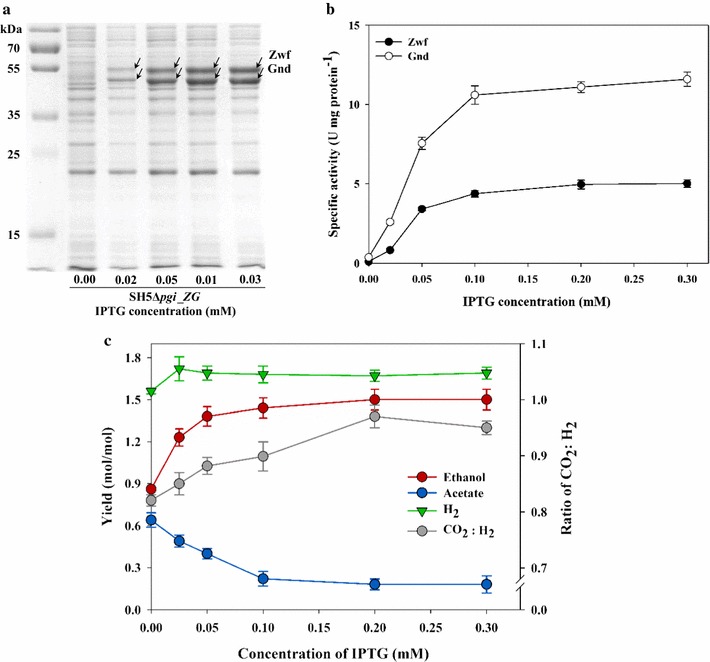
Effect of inducer concentration on co-production of H_2_ and ethanol by SH5Δ*pgi*_ZG. **a** SDS-PAGE analysis of Zwf (55 kDa) and Gnd (51 kDa) in soluble fraction. **b** Enzyme activity of Zwf and Gnd of SH5Δ*pgi*_ZG induced with different IPTG concentrations. **c** Metabolite yields and ratios of CO_2_ to H_2_ evolution of SH5Δ*pgi*_ZG induced with 0, 0.02, 0.05, 0.1, 0.2, and 0.3 mM IPTG

Although expression of *zwf* and *gnd* greatly improved ethanol production while reducing acetate production, the theoretical maximum yield of ethanol production (1.67 mol mol^−1^ glucose) was not achieved. To determine if ethanol production could be further improved, the expression levels of the two major enzymes, Zwf and Gnd, were varied by varying the IPTG concentration within the 0–0.3 mM range during the cultivation of SH5Δ*pgi*_*ZG* (Fig. 3). With increasing IPTG concentration, production of Zwf and Gnd proteins (as analyzed by SDS-PAGE) and their enzymatic activities (from crude cell extract) increased almost linearly to 0.3 mM IPTG. Accordingly, while acetate production decreased, ethanol production and the CO_2_/H_2_ ratio gradually increased. This indicated that when Gnd activity is enhanced, the flux through the PP pathway becomes more prominent than the ED pathway [16]. However, the ethanol yield increase, or the decrease in acetate production, almost halted at ~0.1 mM IPTG; moreover, even at the highest IPTG concentration tested in this study, 0.3 mM, about 0.2 mol acetate mol^−1^ glucose was produced. This observation suggests that achieving the theoretical maximum ethanol production level is impossible by simply controlling the expression level of the current Zwf and Gnd, because the carbon flux through the PP pathway cannot be sufficiently enhanced. Here, it seems that either the enzyme kinetics or the competition between Gnd and Edd determines the carbon distribution at the 6PG node, the amount of NAD(P)H production, and/or the ethanol production yield. In any case, we could achieve high co-production yields, 1.69 mol H_2_ mol^−1^ glucose, and 1.50 mol ethanol mol^−1^ glucose, with SH5Δ*pgi_ZG* at 0.2 mM IPTG.

Next, RT-PCR analysis was performed to examine the changes in the gene expressions of the major glycolytic enzymes in SH5Δ*pgi*_*Z* and SH5Δ*pgi*_*ZG* (Table 3; Additional file 2: Appendix A). In both strains, transcription of *pgi* was not observed, confirming the removal of *pgi*. The *zwf* and/or *gnd* genes were highly expressed in SH5 Δ*pgi*_*Z* and/or Δ*pgi*_*ZG*, and their levels increased when the cells were induced with higher IPTG concentrations. We noticed that the expressions of *pfkA* and *gapA* increased as Zwf and Gnd were more expressed. We attributed these increased transcriptions to enhanced PP pathway flux, because the PP pathway is linked with the EMP pathway at F6P (*pfkA*) and glyceraldehyde-3-phosphate (G3P; *gapA*) nodes, and, with the ED pathway at G3P and pyruvate nodes [27]. Interestingly, transcription of *adhE* also significantly increased when the PP pathway was activated. In this regard, it has been reported that *adhE* transcription increases when the intracellular NAD(P)H level increases, and that this can enhance ethanol production [28]. Surprisingly in the present results, the expression level of *udhA*, encoding NADH:NADPH transhydrogenase that converts NADPH to NADH, did not change upon activation of the PP pathway. This raises the important question of whether NADPH is used directly or after being converted to NADH in the production of ethanol from acetyl-CoA (see “Limitations on PP pathway operation under anaerobic condition and expression of transhydrogenase”).

**Table 3:** Relative transcription levels of key glycolytic enzymes in SH5Δ*pgi*_*Z* and SH5Δ*pgi*_*ZG*

Gene	SH5Δ*pgi*_*Z* (0.1 mM)	SH5Δ*pgi*_*ZG* (0.1 mM)	SH5Δ*pgi*_*ZG* (0.2 mM)
*pgi*	0.00	0.00	0.00
*zwf*	2090.91 ± 146.36	2693.55 ± 212.52	8001.77 ± 400.09
*gnd*	6.03 ± 0.21	2155.47 ± 73.18	6039.95 ± 259.79
*pfkA*	2.16 ± 0.11	3.59 ± 0.06	5.48 ± 0.13
*pfkB*	2.59 ± 0.13	1.29 ± 0.03	1.19 ± 0.03
*gapA*	34.12 ± 1.54	44.01 ± 0.96	80.95 ± 3.24
*edd*	1.41 ± 0.02	1.09 ± 0.01	0.87 ± 0.01
*tktA*	12.20 ± 0.24	5.72 ± 0.12	7.28 ± 0.16
*pflB*	42.99 ± 1.38	21.51 ± 0.65	37.04 ± 0.74
*fhlA*	0.38 ± 0.01	0.52 ± 0.01	0.43 ± 0.01
*udhA*	0.45 ± 0.01	0.55 ± 0.01	0.28 ± 0.01
*adhE*	4.51 ± 0.05	7.22 ± 0.12	13.76 ± 0.47

The result was from three individual experiment repeats

*rpoD* was used as the endogenous control and the transcriptional level of rpoD was considered as 1

### Construction and characterization of SH5*ΔpgiΔedd*, the strain using PP pathway as sole glycolytic route

The improved activity of Zwf and Gnd could not completely eliminate acetate production in SH5Δ*pgi*_*ZG*. Therefore, to make the PP pathway the sole glycolytic route, the ED pathway was blocked by disruption of the *edd* and *eda* genes from SH5Δ*pgi*. The resultant SH5 Δ*pgi*Δ*edd* strain could grow on glucose under aerobic conditions but not at all under anaerobic conditions, even after overexpression of Zwf and Gnd (see Additional file 1: Fig. S2). This result, albeit disappointing, confirmed that the ED pathway is functioning in the SH5Δ*pgi*_*ZG* strain and metabolizing a portion of the glucose.

The inability of SH5Δ*pgi*Δ*edd*_*ZG* to grow on glucose under anaerobic condition is attributed to redox imbalance. According to the carbon and energy balance, when one glucose is fully metabolized through the PP pathway, 0.33 NADPH is generated along with 1.67 H_2_ and 1.67 ethanol [13] (Fig. 1b). The supplementation of yeast extract in the medium could have worsen the redox imbalance because it contains complex amino acids and some carbohydrates which can contribute to the regeneration of NAD(P)H. If excess NADPH is accumulated, the PP pathway will be blocked along with termination of cell growth. To prove this hypothesis, two experiments were carried out. First, SH5Δ*pgi*Δ*edd* _*ZG* was grown on gluconate as the gluconate is more oxidized than glucose, and so no excess NADPH is accumulated. As expected, SH5Δ*pgi*Δ*edd*_*ZG* could grow on gluconate, though the rate is very slow (see Additional file 1: Fig. S2). In the course of that growth, ethanol and acetate were produced at yields of 0.79 and 0.84 mol mol^−1^, respectively, which makes the gluconate metabolism redox-balanced. In the second experiment, SH5Δ*pgi*Δ*edd*_*ZG* was grown on glucose but in the presence of nitrate (Additional file 1: Fig. S3). Nitrate can be used as an external electron acceptor and regenerate NAD(P)^+^ under anaerobic conditions [29]. The results showed that the addition of nitrate recovered the growth of SH5Δ*pgi*Δ*edd* even without the expression of Zwf and Gnd (Additional file 1: Fig. S3). The overexpression of Zwf and Gnd in the presence of nitrate increased the glucose consumption. However, neither H_2_ nor ethanol was produced; instead, acetate was the sole metabolite. In the presence of nitrate, *E. coli* oxidizes NAD(P)H to reduce nitrate and produce ATP. In summary, these two experiments strongly suggest that the redox imbalance and/or excessive NADPH generated by the PP pathway prevented SH5Δ*pgi*Δ*edd*_*ZG* growth on glucose under anaerobic conditions.

It is possible to determine the minimal glucose flux to the ED pathway allowing for redox-balanced glucose metabolism using the carbon balance equation of PP and ED pathway (Fig. 1b) (see Additional file 1: Fig. S4). When NAD(P)H used for cell growth is ignored, the estimated minimal flux ratio of the ED pathway (i.e., ED flux/sum of PP and ED fluxes) is 0.14. If the minimal flux ratio of the ED pathway is below 0.14, the production and consumption of NAD(P)H cannot be matched by the production of ethanol and acetate. On the other hand, at any flux ratios above 0.14, combined production of acetate and ethanol makes the glucose metabolism redox-balanced and allows cells to grow.

### Limitations on PP pathway operation under anaerobic condition and expression of transhydrogenase

According to the redox-balance analysis results plotted in Additional file 1: Fig. S4, the current SH5Δ*pgi*_*ZG* had a higher ED flux (~0.32) than the ideal case (0.14). We speculate that despite high expression by the multi-copy plasmid, the enzymatic activities of Zwf and Gnd were low under the physiological conditions, and that this might be the reason why the flux ratio to the PP pathway did not increase above the 0.1 mM IPTG shown in Fig. 3. It is known that Zwf and Gnd of *E. coli* are almost exclusively NADP^+^ dependent and that their enzymatic activities are highly inhibited by NADPH [24, 30] (see Additional file 1: Table S2). Because the roles of Zwf and Gnd are so important, we cloned and characterized these enzymes from our own host *E. coli* BW25113 (Additional file 1: Fig. S5, Additional file 1: Table S2). The enzymes were expressed with a C-terminal His-tag and characterized after purification by Ni–NTA chromatography. Both of them were shown to be strictly dependent on NADP^+^, and no activity was observed with NAD^+^ as the cofactor. The specific activities and *K*_m_ values of the purified Zwf and Gnd were similar to those that have been reported [30] (Additional file 1: Table S2). Additionally, we found that the two enzymes were inhibited by NADPH at similar levels (*K*_*i*_ = ~ 40 μM) but not at all by NADH.

The problem associated with high intracellular NADPH concentration and consequent inhibition on Zwf and Gnd can be solved in two ways: by (1) reducing the NADPH concentration and/or (2) employing less NADPH-sensitive enzymes. To explore the first approach, we overexpressed UdhA, the soluble transhydrogenase for the conversion of NADPH to NADH. *E. coli* strains have two transhydrogenases, one soluble (*udhA*) and the other membrane bound (*pntAB*) [31]. Although both enzymes work reversibly, the former mainly catalyzes the reaction for the conversion of NADPH to NADH, and the latter, the reverse reaction [31]. To our disappointment, even after the overexpression of UdhA under a strong *tac* promoter, no improvement in ethanol production was observed in SH5Δ*pgi*_*ZG* (Additional file 1: Fig. S6). Further, deletion of both *udhA* and *pntA* from SH5Δ*pgi*_*ZG* did not affect cell growth or metabolite formation: SH5Δ*pgi*Δ*udhA*Δ*pntA*_*ZG* grew similar to SH5Δ*pgi*_*ZG* and produced similar amounts of H_2_, ethanol, and acetate (Additional file 1: Fig. S6). These results are puzzling, because they indicate that the roles of the two transhydrogenases are negligible in glucose metabolism, and also that ethanol production in SH5Δ*pgi*_*ZG* and other derived strains might be NADPH dependent. In any case, it is clear that overexpression of transhydrogenases cannot be the solution to the problem that necessitates reduction of intracellular NADPH levels and/or enhancement of carbon flux through the PP pathway.

### Use of heterologous *zwf* and *gnd*

In another attempt to improve the carbon flux to the PP pathway, we overexpressed heterologous Zwf and Gnd which are less inhibited by NADPH. If such enzymes can use NAD^+^ as the cofactor, the reduction of the intracellular NADPH level would be also expected. For the Zwf and Gnd reported in the literature and enzyme databases, cofactor specificity, activity, and NADPH-dependent inhibition were analyzed and compared (Additional file 1: Table S2). The Zwf from *Zymomonas mobilis* (Z_Z_) had an approximately sevenfold higher activity than that of *E. coli* (Z_E_; note that the subscript ‘_E_’ was added to avoid confusion) [32]. Furthermore, it could use both NAD^+^ (*K*_m_, 210 µM) and NADP^+^ (*K*_m_, 40 µM), though preferring the latter more. The Zwf from *Leuconostoc mesenteroides* (Z_L_) also showed a sevenfold higher activity than that of Z_E_, and could use NAD^+^ as a cofactor, having a higher affinity (*K*_m_, 106 µM) than Z_Z_ [33, 34]. Interestingly, the Gnd from *Gluconobacter oxydans* (G_G_) showed a higher affinity to NAD^+^ (*K*_m_, 64 µM) than to NADP^+^ (*K*_m_, 440 µM), whereas Gnd from *Corynebacterium glutamicum* (G_C_) showed a fivefold higher activity than that of *E. coli* (G_E_), though its use of NAD^+^ as a cofactor is not known [24, 35].

Next, different recombinant plasmids were constructed for the expression of heterologous Zwf (Z_E_, Z_L_, Z_Z_) and Gnd (G_E_, G_G_, G_C_) in various combinations and then introduced to SH5Δ*pgi* to generate eight recombinant strains (Table 4). These recombinant SH5Δ*pgi* strains were studied for the growth and production of H_2_ and ethanol with glucose as the carbon source. Except for SH5Δ*pgi*_*Z*_*E*_ *G*_*C*_ and SH5Δ*pgi*_*Z*_*E*_ *G*_*G*_, the other (six) strains grew well and produced H_2_ similar to that of SH5Δ*pgi*_*Z*_*E*_ *G*_*E*_ at 1.6 ± 0.1 mol mol^−1^ glucose. Those six recombinant strains also generated similar or higher amounts of ethanol than SH5Δ*pgi*_*ZG*; among them, the SH5Δ*pgi*_*Z*_*L*_ *G*_*G*_ strain, which expresses Zwf of *L. mesenteroides* and Gnd of *G. oxydans*, showed the highest ethanol production yield at 1.62 mol mol^−1^ glucose as well as the lowest acetate production yield at ~0.06 mol mol^−1^ glucose. It was considered that the high activity and dual-cofactor specificity of Z_L_ along with the NAD^+^ dependence of G_G_ were the main reasons for the improved performance of SH5Δ*pgi*_*Z*_*L*_ *G*_*G*_. The flux ratio of the ED pathway in this strain, moreover, was estimated to be close to the thermodynamically allowed lowest level, 0.14 (see Additional file 1: Fig. S4).

**Table 4:** Co-production by recombinant SH5Δ*pgi* and SH5Δ*pgi*Δ*edd* strains overexpressing heterologous Zwf and Gnd

Strains	Relative growth rate	Yield of metabolites (mol mol^−1^)	
		Ethanol	Acetate
Δ*pgi*_ZG	+++	1.44 ± 0.07	0.22 ± 0.03
Δ*pgi*_Z_L_G_E_	++	1.48 ± 0.04	0.25 ± 0.02
Δ*pgi*_Z_Z_G_E_	+++	1.49 ± 0.06	0.32 ± 0.01
Δ*pgi*_Z_E_G_C_	+	1.35 ± 0.05	0.37 ± 0.01
Δ*pgi*_Z_L_G_C_	+++	1.46 ± 0.06	0.26 ± 0.01
Δ*pgi*_Z_Z_G_C_	+++	1.32 ± 0.07	0.49 ± 0.01
Δ*pgi*_Z_E_G_G_	+	1.52 ± 0.09	0.22 ± 0.02
Δ*pgi*_Z_L_G_G_	++	1.62 ± 0.06	0.06 ± 0.01
Δ*pgi*_Z_Z_G_G_	++	1.46 ± 0.07	0.35 ± 0.01
Δ*pgi*Δ*edd*_Z_L_G_G_^a^	++	1.01 ± 0.03	0.40 ± 0.01

The result was from three individual experiment repeats

^a^Δ*pgi*Δ*edd*_Z_L_G_G_ was grown on gluconate

The same plasmid expressing both Zwf of *L. mesenteroides* and Gnd of *G. oxydans* (pLmZ-GoG) was introduced to SH5Δ*pgi*Δ*edd* to determine if these highly efficient Zwf and Gnd can enable its anaerobic growth. As expected, the resulting recombinant SH5Δ*pgi*Δ*edd*_*Z*_*L*_ *G*_*G*_ could not grow anaerobically with glucose as the carbon source. This confirmed that redox imbalance does not permit use of the PP pathway as the sole glycolytic route of glucose metabolism under anaerobic conditions. The same SH5Δ*pgi*Δ*edd*_*Z*_*L*_ *G*_*G*_ strain was also cultured on gluconate as the carbon source. In fact, it could grow much better than SH5Δ*pgi*Δ*edd*_*ZG* (Additional file 1: Fig. S2), producing more ethanol (1.01 vs. 0.79 mol mol^−1^) and less acetate (0.40 vs. 0.85 mol mol^−1^). This result confirmed once again that the highly efficient Zwf (Z_L_) and Gnd (G_G_) can effectively activate the PP pathway.

The Δ*pfkA* strains could grow well after long adaptation to anaerobic growth [14] and produced good amounts of H_2_ and ethanol (~1.7 mol H_2_ mol^−1^ and ~1.40 mol ethanol mol^−1^) when Zwf and Gnd, the key enzymes of the PP pathway, were overexpressed. However, due to the active expression of *pfkB* which encodes for the isozyme of PfkA, up to 30% of glucose was metabolized through the EMP pathway in the Δ*pfkA* strains and substantial amount of acetate was produced (~0.15 mol mol^−1^). On the other hand, the Δ*pgi* strains led us to understand the metabolic hurdles in achieving theoretical maximum yield. In addition, the usage of efficient Zwf and Gnd enzymes in the Δ*pgi* strains led us to successfully achieve the theoretical maximum yield of H_2_ and ethanol (~1.6 mol mol^−1^ each).

## Conclusions

The *E. coli*Δ*pgi* mutant could grow on glucose under anaerobic conditions when Zwf was overexpressed, but when both Zwf and Gnd were overexpressed, diversion of the carbon flux through the PP pathway and efficient co-production of H_2_ and ethanol were possible. Operation of the PP pathway as the sole glycolytic route for glucose under anaerobic conditions, however, was not possible, due to the redox imbalance. When the EMP pathway was blocked by *pgi* deletion, there existed, for the ED pathway, a critical flux ratio (0.14) above which cell growth was possible. The flux distribution between the PP and ED pathways at the 6-phosphogluconate node, and the co-production yield of H_2_ and ethanol, were determined by the characteristics of Zwf and Gnd. When *zwf* from *L. mesenteroides* and *gnd* from *G. oxydans*, both of which use NAD^+^ and NADP^+^ as cofactors and are less inhibited by NADPH, were employed, the best co-production yield of H_2_ and ethanol (1.74 mol H_2_ mol^−1^ glucose; 1.62 mol ethanol mol^−1^ glucose), close to the theoretical maximum values (1.67 mol mol^−1^ glucose for each), resulted. Activation of the PP pathway, as presented in this work, will be found to be useful for developing efficient biocatalysts for other biofuels and biochemicals that require additional reducing power and need to be produced under anaerobic conditions.

## Additional files

**Additional file 1: Table S1.** Details of Zwf and Gnd used in this study. **Table S2.** Kinetic properties of Zwf and Gnd used in this study. **Figure S1.** Adaptive evolution for anaerobic growth of SH5Δ*pgi* with glucose as substrate. **Figure S2.** Anaerobic growth of recombinant SH5Δ*pgi*Δ*edd* strains on glucose (Glu) and gluconate (Gln). Refer to Table 1 for the genotype of each strain. **Figure S3.** Growth and acetate production yield of SH5Δ*pgi*Δ*edd* and SH5Δ*pgi*Δ*edd_ZG* on glucose in the presence of nitrate. **Figure S4.** Theoretical prediction of relation between dependence on ED pathway and ethanol and acetate production. Redox-imbalanced region denotes the production of excess NADPH than pyruvate. **Figure S5.** SDS-PAGE analyses of Zwf (55 kDa) and Gnd (51 kDa) in soluble fraction SH5_pDK7_*zwf* (Lane 1) and SH5_pDK7_*gnd* (Lane 2) and purified Zwf (Lane 3) and Gnd (Lane 4) by Ni–NTA chromatography. **Figure S6.** Growth and metabolites production yield of SH5Δ*pgi_ZGU* and SH5Δ*pgi*Δ*udhA*Δ*pntA_ZG* on glucose.

**Additional file 2: Appendix A.** Raw data of RT-PCR analysis.

## Abbreviations

CO_2_: carbon dioxide
ED: Entner–Doudoroff
EMP: Embden–Meyerhof–Parnas
H_2_: hydrogen
IPTG: isopropyl-β-d-thiogalactopyranoside
OD_600_: optical density
Pgi: phosphoglucose isomerase
PP: pentose-phosphate
