# Supplementary material for: Co-production of hydrogen and ethanol from glucose in Escherichia coli by activation of pentose-phosphate pathway through deletion of phosphoglucose isomerase (pgi) and overexpression of glucose-6-phosphate dehydrogenase (zwf) and 6-phosphogluconate dehydrogenase (gnd)
Source: Biotechnol Biofuels. 2017 Mar 29;10:85. doi: 10.1186/s13068-017-0768-2 (PMC5372246; doi:10.1186/s13068-017-0768-2)
Supplement: Supplementary file 1 — Additional file 1: Table S1. Details of Zwf and Gnd used in this study. Table S2. Kinetic properties of Zwf and Gnd used in this study. Figure S1. Adaptive evolution for anaerobic growth of SH5Δpgi with glucose as substrate. Figure S2. Anaerobic growth of recombinant SH5ΔpgiΔedd strains on glucose (Glu) and gluconate (Gln). Refer to Table 1 for the genotype of each strain. Figure S3. Growth and acetate production yield of SH5ΔpgiΔedd and SH5ΔpgiΔedd_ZG on glucose in the presence of nitrate. Figure S4. Theoretical prediction of relation between dependence on ED pathway and ethanol and acetate production. Redox-imbalanced region denotes the production of excess NADPH than pyruvate. Figure S5. SDS-PAGE analyses of Zwf (55 kDa) and Gnd (51 kDa) in soluble fraction SH5_pDK7_zwf (Lane 1) and SH5_pDK7_gnd (Lane 2) and purified Zwf (Lane 3) and Gnd (Lane 4) by Ni–NTA chromatography. Figure S6. Growth and metabolites production yield of SH5Δpgi_ZGU and SH5ΔpgiΔudhAΔpntA_ZG on glucose. [file 13068_2017_768_MOESM1_ESM.docx]

Table S1: Details of Zwf and Gnd used in this study

| **Source** | **GenBank**  **Accession no.** | **Molecular weight (kDa)*** | **Primer sequence** |
| --- | --- | --- | --- |
| **Zwf** | | | |
| *Escherichia coli* | AIN32292 | 55.7 | FP: CAA TCT AGA TAA GGA GAT ATA CCA TGG CGG TAA CGC AAA CAG CCC AG  RP: CAA AAG CTT TTA CTC AAA CTC ATT CCA GGA ACG ACC |
| *Leuconostoc mesenteroides^#^* | WP_002815576 | 54.4 | FP: CAA TCT AGA TAA GGA GAT ATA CCA TGG TTA GCG AGA TTA AGA CCC TGG  RP: CAA GTC GAC TTA GTG GTG GTG GTG GTG ATG ACC TTT AAAC |
| *Zymomonas mobilis^#^* | WP_011240288 | 53.7 | FP: CAA TCT AGA TAA GGA GAT ATA CCA TGA CCA ACA CCG TGA GCA CCA TG  RP: CAA GTC GAC TTA GTG GTG GTG GTG GTG ATG GTC ATA CC |
| **Gnd** | | | |
| *Escherichia coli* | AIN32448 | 51.4 | FP: CAA GGA TCC TAA GGA GAT ATA CCA TGT CCA AGC AAC AGA TCG GCG TAG  RP: CAAT CTA GAT TAA TCC AGC CAT TCG GTA TGG AAC AC |
| *Corynebacterium glutamicum^#^* | NP_600669 | 51.7 | FP: CAA GGA TCC TAA GGA GAT ATA CCA TGA CCA ACG GTG ACA ACC TGG CGC  RP: CAA TCT AGA TTA GTG GTG GTG GTG GTG GTG CGC TTC CAC |
| *Gluconobacter oxydans^#^* | WP_011253227 | 35.7 | FP: CAA GGA TCC TAA GGA GAT ATA CCA TGC GTA TTG GTA TCA TTG GTC TGG  RP: CAA TCT AGA TTA GTG GTG GTG GTG GTG GTG TTT CTT C |

* Molecular weight was predicted using ExPASy Compute pI/Mw tool

^#^ The genes were codon-optimized for increased soluble expression in *E. coli* and synthesized.

Table S2: Kinetic properties of Zwf and Gnd used in this study

| **Organism** | **Activity (U/mg)** | **Cofactor** | ***K_m_* (µM)** | **Inhibitor** | ***K_i_* (µM)** | **Reference** |
| --- | --- | --- | --- | --- | --- | --- |
| **Glucose-6-phosphate dehydrogenase (Zwf)** | | | | | | |
| *E. coli** | 75 | NADP^+^ | 15 | NADPH | 40 | This study, Olavarria et al. [30] |
| *Z. mobilis* | 680 | NAD^+^ | 210 | N/A | N/A | Scopes et al. [32] |
|  | 390 | NADP^+^ | 40 | N/A | N/A |  |
| *L. mesenteroides* | 715 | NAD^+^ | 106 | NADH | 1000 | Vought et al. [34] |
|  | N/A | NADP^+^ | 8 | NADPH | 38 |  |
| **6-Phosphogluconate dehydrogenase (Gnd)** | | | | | | |
| *E. coli** | 31 | NADP^+^ | 40 | NADPH | 40 | This study |
| *G. oxydans* | 41 | NAD^+^ | 64 | N/A | N/A | Tonouchi et al. [35] |
|  | 41 | NADP^+^ | 440 | N/A | N/A |  |
| *C. glutamicum** | 143 | NADP^+^ | 40 | NADPH | 100 | Moritz et al. [24] |

N/A: Information not available

*Zwf and Gnd from *E. coli* and Gnd from *C. glutamicum* could not use NAD^+^ as cofactor.


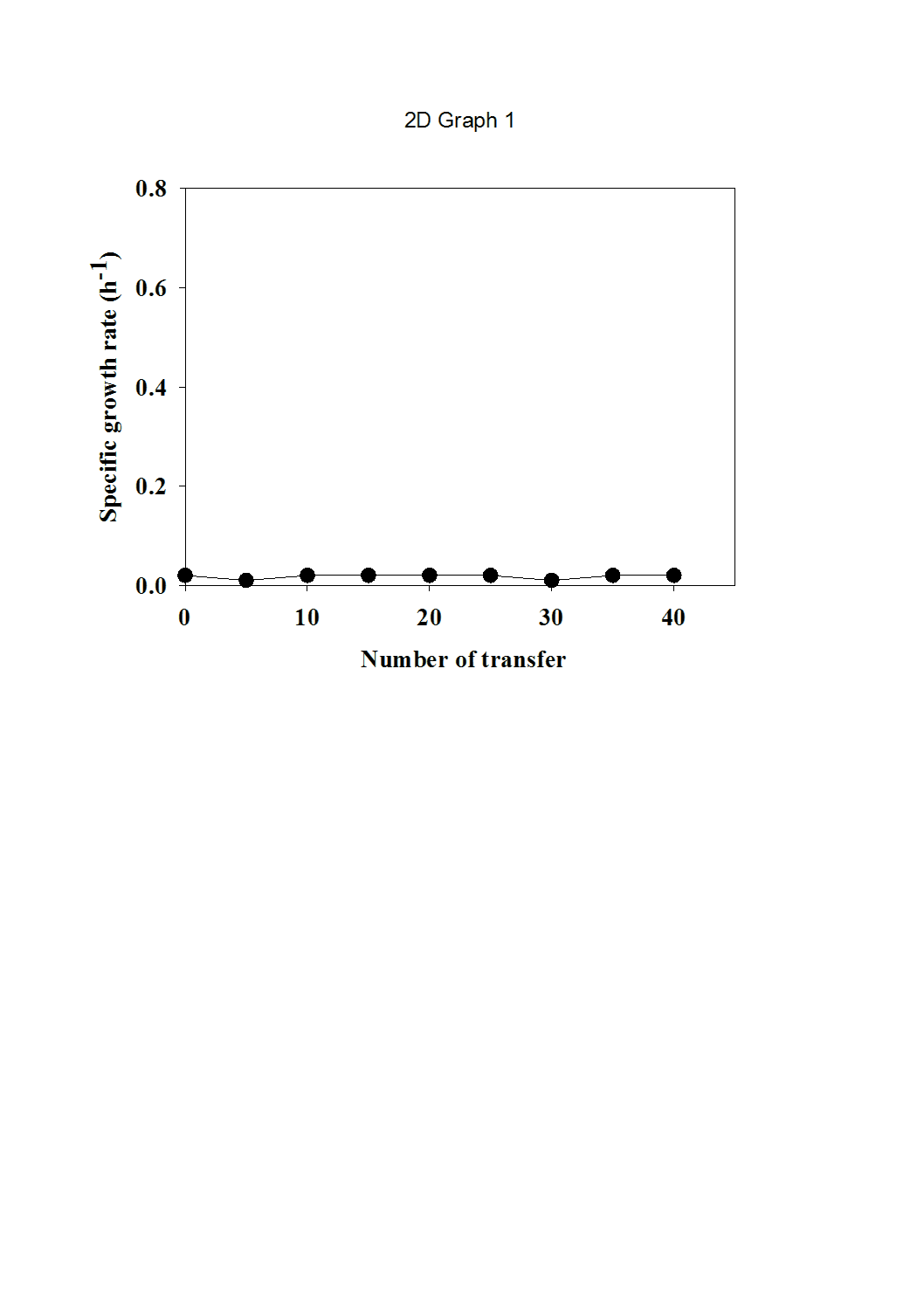


Figure S1: Adaptive evolution for anaerobic growth of SH5Δ*pgi* with glucose as substrate.





Figure S2: Anaerobic growth of recombinant SH5Δ*pgi*Δ*edd* strains on glucose (Glu) and gluconate (Gln). Refer to Table 1 for the genotype of each strain.


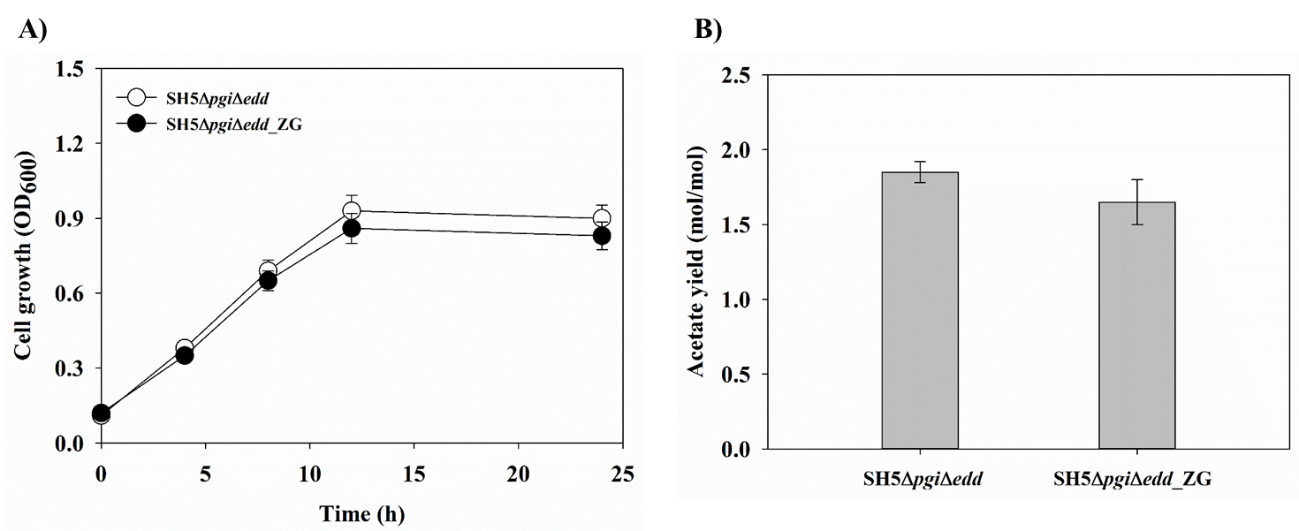


Figure S3: Growth and acetate production yield of SH5Δ*pgi*Δ*edd* and SH5Δ*pgi*Δ*edd_ZG* on glucose in the presence of nitrate.

Figure S4: Theoretical prediction of relation between dependence on ED pathway and ethanol and acetate production. Redox-imbalanced region denotes the production of excess NADPH than pyruvate.


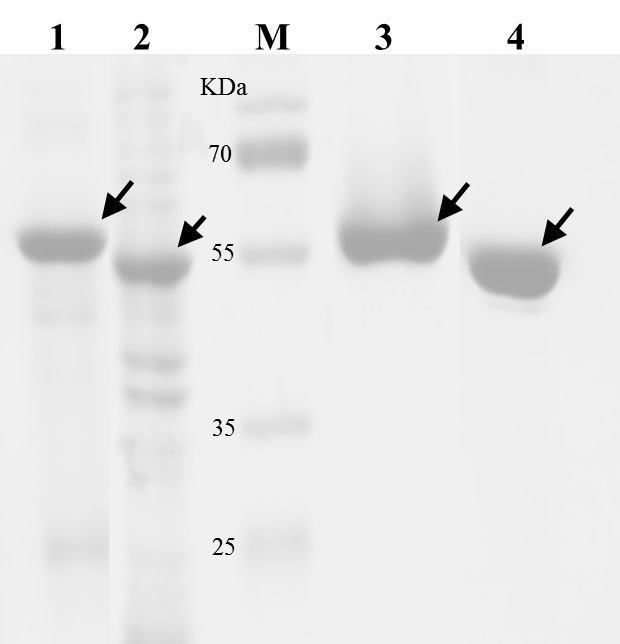


Figure S5: SDS-PAGE analyses of Zwf (55 kDa) and Gnd (51 kDa) in soluble fraction SH5_pDK7_*zwf* (Lane 1) and SH5_pDK7_*gnd* (Lane 2) and purified Zwf (Lane 3) and Gnd (Lane 4) by Ni-NTA chromatography.


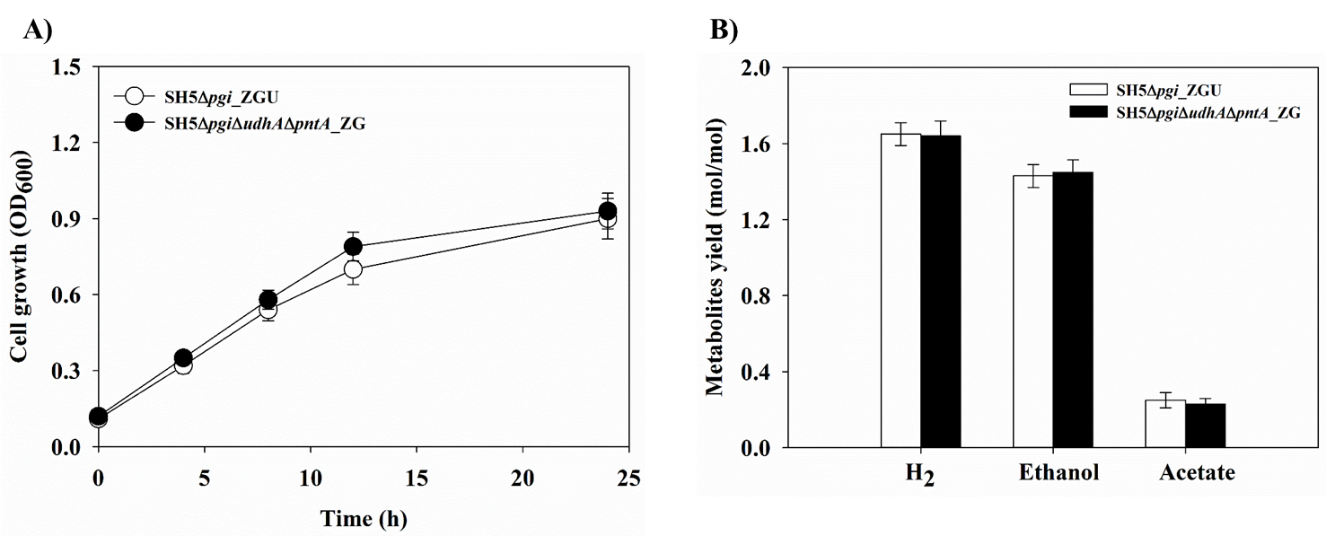


Figure S6: Growth and metabolites production yield of SH5Δ*pgi_ZGU* and SH5Δ*pgi*Δ*udhA*Δ*pntA_ZG* on glucose.
